# Mechanism of Bile Acid in Regulating Platelet Function and Thrombotic Diseases

**DOI:** 10.1002/advs.202401683

**Published:** 2024-06-23

**Authors:** Xianghui Zhou, Xin Zhou, Zhao Zhang, Ruirui Zhu, Meng Lu, Keyu Lv, Chao Fang, Zhangyin Ming, Zhipeng Cheng, Yu Hu

**Affiliations:** ^1^ Department of Hematology Union Hospital Tongji Medical College Huazhong University of Science and Technology Wuhan 430022 China; ^2^ Department of Stomatology Union Hospital Tongji Medical College Huazhong University of Science and Technology Wuhan 430022 China; ^3^ Department of Cardiology Union Hospital Tongji Medical College Huazhong University of Science and Technology Wuhan 430030 China; ^4^ Department of Pharmacology School of Basic Medicine Tongji Medical College of Huazhong University of Science and Technology Wuhan 430030 China

**Keywords:** atherosclerosis, bile acid, platelet, thrombosis

## Abstract

Platelets play a key role in physiological hemostasis and pathological thrombosis. Based on the limitations of current antiplatelet drugs, it's important to elucidate the mechanisms of regulating platelet activation. In addition to dissolving lipid nutrients, bile acids (BAs) can regulate platelet function. However, the specific mechanisms underlying BAs‐mediated effects on platelet activation and thrombotic diseases remain unknown. Therefore, the effects of BAs on platelets and intracellular regulatory mechanisms are explored. It is showed that the inhibitory effect of secondary BAs is more significant than that of primary BAs; lithocholic acid (LCA) shows the highest inhibitory effect. In the process of platelet activation, BAs suppress platelet activation via the spleen tyrosine kinase (SYK), protein kinase B (Akt), and extracellular signal‐regulated kinase1/2 (Erk1/2) pathways. Nck adaptor proteins (NCK1) deficiency significantly suppress the activity of platelets and arterial thrombosis. Phosphorylated proteomics reveal that LCA inhibited phosphorylation of syntaxin‐11 at S80/81 in platelets. Additional LCA supplementation attenuated atherosclerotic plaque development and reduced the inflammation in mice. In conclusion, BAs play key roles in platelet activation via Syk, Akt, ERK1/2, and syntaxin‐11 pathways, which are associated with NCK1. The anti‐platelet effects of BAs provide a theoretical basis for the prevention and therapy of thrombotic diseases.

## Graphical abstract

Bile acids play critical roles in platelet activation via Syk, PLCγ2, PI3K, Akt, and ERK1/2 pathway, which is also associated with Syntaxin‐11 and NCK1. Additional Bile acids (LCA) supplementation attenuated atherosclerotic plaque development and reduce the inflammation in mice. These findings provide important insights for exploring bile acids and their related mechanisms in the treatment of atherosclerosis and thrombosis.

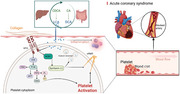

## 1 Introduction

The development of arterial thrombotic diseases, such as acute coronary syndrome (ACS) and cerebral ischemic stroke, involves platelet activation and deformation along with cross‐linking with leukocytes and red blood cells which accumulate at the site of vessel injury, thereby blocking blood flow.^[^ ^1^, ^2^ ^]^ Increased platelet reactivity and thrombosis risk are associated with pro‐inflammatory pathological conditions, such as atherosclerosis (AS), immunological derangement, dyslipidemia, and diabetes.^[^ ^3^, ^4^, ^5^, ^6^ ^]^ However, the mechanism of abnormal platelet activity and pathological thrombosis has not been fully elucidated, which increases the importance of studying these factors.

Over the past few decades, great progress has been achieved in antiplatelet drug development, and several drugs have been used in the clinical treatment of cardiovascular diseases, including cyclooxygenase inhibitor (aspirin), P2Y12 antagonists (ticlopidine, clopidogrel, and prasugrel), phosphodiesterase/adenosine reuptake inhibitors (cilostazol and dipyridamole), glycoprotein IIb/IIIa (αIIbβ3) antagonists (abciximab, integrilin, and tirofiban) and protease‐activated receptor 1 antagonists (vorapaxar and atopaxar).^[^ ^7^ ^]^ However, the use of these first‐line antiplatelet agents is limited by the lack of clinical efficacy and high rates of bleeding complications.^[^ ^7^ ^]^ To address these issues, innovative approaches have been developed based on the differences in signal transduction mechanisms involved in physiological hemostasis and atherosclerotic thrombosis. New antagonists for platelet surface receptors^[^ ^8^, ^9^, ^10^, ^11^, ^12^ ^]^ and intraplatelet signaling proteins have been developed.^[^ ^13^, ^14^, ^15^ ^]^ Several inhibitors have been clinically evaluated, and certain preliminary results appear promising. However, most antiplatelet agents are terminated in clinical trials based on poor specificity and side effects such as bleeding. Therefore, understanding the molecular mechanisms that regulate platelet activation and thrombosis is essential for establishing new targets.

Bile acids (BAs) are products of cholesterol catabolism, and their use has attracted increasing attention owing to various bioactivities.^[^ ^16^ ^]^ They are synthesized in the liver, in which primary BAs are transported to the biliary system and eventually discharged into the small intestine. Enzymes produced by intestinal microbiota modify primary BAs to form secondary BAs, including deoxycholic acid (DCA) and lithocholic acid (LCA).^[^ ^17^, ^18^ ^]^ Subsequently, BAs can be redistributed through the enterohepatic circulation, and this dynamic process maintains a balance in the concentrations of BAs distributed to their physiologically active sites.^[^ ^19^ ^]^

Several studies have shown that BAs exert an anti‐inflammatory effect, which is characterized by significant remission of rheumatic symptoms in patients with jaundice exhibiting elevated levels of circulating BAs.^[^ ^20^ ^]^ In addition to the anti‐inflammatory effect of BAs, the activation of BA receptors can exert anti‐inflammatory effects in AS. Pols reported that Takeda G‐protein‐coupled receptor 5 (TGR5) activation reduces macrophage inflammation and lipid absorption, thereby exerting anti‐AS effects.^[^ ^21^ ^]^ Furthermore, farnesoid X receptor (FXR) activation exerts anti‐atherosclerotic effects, which may derive from a combined modulation of liver lipid metabolism, macrophage cholesterol uptake and efflux, and inflammation.^[^ ^22^, ^23^ ^]^ The dual activation of FXR and TGR5 can also reduce the levels of circulating lipids and inflammation via protein kinase A/nuclear factor‐kappa B signal transduction.^[^ ^24^ ^]^ Therefore, BAs may function as natural ligands of FXR and TGR5 and they may also have anti‐inflammatory and anti‐atherogenic effects. In addition to FXR and TGR5, BAs also correspond to other nuclear and membrane receptors, such as vitamin D3 receptor (VDR), constitutive androstane receptor (CAR), sphingosine 1‐phosphate receptor 2 (S1PR2), formyl‐peptide receptors (FPRs), and muscarinic acetylcholine receptors (mAChRs). Previous findings suggest that BAs exert anti‐inflammatory effects through FPRs. However, in this field, studies on the anti‐inflammatory and anti‐atherosclerosis effects of BAs are limited, which is worthy of further exploration.

In the past few years, the relationship between BAs and platelet activation has been evaluated,^[^ ^25^ ^]^ whereas the underlying mechanisms of BAs in platelet activation remain unclear. Baele et al. showed that bile salts are capable of inhibiting platelet aggregation in vitro.^[^ ^26^ ^]^ For bile acid receptors, studies have found that the FXR ligand can inhibit platelet activation, which may be related to promoting coated platelet formation.^[^ ^27^, ^28^ ^]^ However, the regulatory effects of different types of BAs on platelet activation, thrombotic diseases, and intracellular mechanisms have not been fully clarified, and further exploration is still needed.

In the present study, we explored the regulatory effects of different types of BAs on platelet activation and thrombosis. Phosphorylated proteomics was used to identify a series of modified proteins in platelet activation under LCA, such as syntaxin‐11, etc. We also demonstrated the role of NCK1 in platelet activation through kinase analysis. Therefore, we constructed NCK1 knockout mice to investigate its role in platelet activation and arteriovenous thrombosis. In addition, we further explored the influence of LCA on the development of arterial thrombotic disease by constructing an atherosclerosis model.

## 2 Results

### 2.1 BAs Inhibited Platelet Aggregation

**Figure 1:**
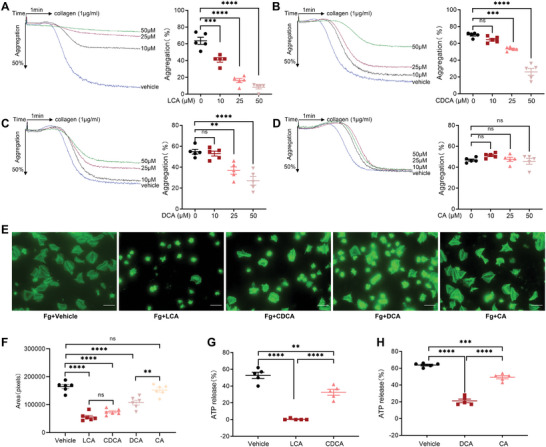
BAs inhibited platelet aggregation, spreading, and ATP Release. A–D) Washed platelets were incubated with LCA, CDCA, DCA, or CA in the presence of 1 mM CaCl_2_ for 5 min, following stimulation with collagen (1 µg mL^−1^). Three concentrations of every BAs were used as indicated; *n* = 5. E,F) Washed platelets were incubated with 50 µm LCA, CDCA, DCA, or CA for spreading on immobilized fibrinogen. E) Representative images and F) quantification of the areas of spreading platelets; *n* = 6. G,H) ATP release of human platelets pretreated with LCA, CDCA, DCA, or CA (25 µm) in response to collagen (1 µg mL^−1^); *n* = 5. Data are presented as the mean ± standard error of mean; one‐way analysis of variance; ^*^ *p* < 0.05, ^**^ *p* < 0.01, ^***^ *p* < 0.001, ^****^ *p* < 0.0001, NS indicates no significance.

We found that LCA, CDCA, and DCA inhibited platelet activation stimulated by collagen (1 µg mL^−1^) within the set concentration gradient (0, 10, 25, and 50 µm). However, CA did not show inhibitory activity under this concentration gradient (**Figure** **1**A–D). Under collagen stimulation, the inhibitory effect of secondary BAs was greater than that of primary BAs. To investigate the effect of BAs on platelets in the presence of other agonists, we adjusted the concentrations of the four BAs. We found that LCA and CDCA inhibited human platelet aggregation stimulated by collagen, thrombin, U46619, and ADP. DCA and CA inhibited platelet aggregation only under stimulation with collagen, U46619, and ADP; however, they had no inhibitory effect on thrombin stimulation at the concentration gradient of 25, 50, and 100um. In addition, BAs did not affect platelet aggregation under the stimulation of AA (Figure S1A–D, Supporting Information). Further, we evaluated the cytotoxic effect of BAs on platelets by measuring the release of LDH. No significant increase in LDH levels was observed in platelet supernatant after BAs administration (Figure S2A–D, Supporting Information), suggesting that BAs have no cytotoxic effect on platelets at our experimental dose. In addition, we found that BAs (LCA (25 µm), CDCA (25 µm), DCA (25 µm), and CA (25 µm)) did not affect phosphatidylserine exposure (Annexin‐V binding) within platelets, as determined by flow cytometry (Figure S2E, Supporting Information). Moreover, LCA itself could not promote platelet aggregation (Supplemental Figure S2F,G).

### 2.2 BAs Inhibited Spreading and ATP Release

The platelet spreading assay showed that LCA, CDCA, and DCA greatly inhibited the spreading of human platelets on fibrinogen when the concentration of BAs was 50 µm, while CA did not (Figure 1E,F). In addition, BAs inhibited ATP release from washed human platelets induced by collagen, and the inhibitory effect of secondary BAs was greater than that of primary BAs (Figure 1G,H).

### 2.3 BAs Inhibited the Downstream Signaling Pathways of GPVI

**Figure 2:**
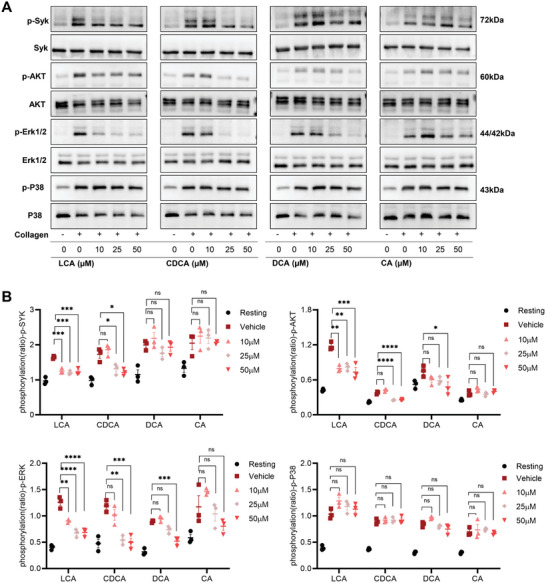
BAs inhibited the downstream signaling pathways of GPVI. A,B) Human platelets were pretreated with LCA, CDCA, DCA, or CA (0, 10, 25, or 50 µm), stimulated with collagen (1 µg mL^−1^), and lysed with lysis buffer for immunoblotting. We used the primary antibodies, including p‐SYK, p‐AKT, p‐ERK1/2, and p‐P38; *n* = 3. Data are presented as the mean ± standard error of mean; one‐way ANOVA; ^*^ *p* < 0.05, ^**^ *p* < 0.01, ^***^ *p* < 0.001, ^****^ *p* < 0.0001, NS indicates no significance.

We demonstrated that BAs inhibited collagen‐stimulated platelet aggregation. When activated, GPVI, the main receptor of collagen on platelets, activates downstream signaling pathways including Protein Kinase C (PKC), phosphatidylinositol‐3‐hydroxykinase (PI3K)/Akt and mitogen activated protein kinase via Src and Syk tyrosine kinase, which could lead to platelet skeleton rearrangement and particle secretion, and eventually platelet activation.^[^ ^29^, ^30^ ^]^ To further understand the mechanism underlying this effect, we examined the effect of BAs on the downstream signaling molecules under collagen stimulation. BAs inhibited the phosphorylation of SYK, ERK1/2, and AKT in platelets. However, the phosphorylation level of P38 was not affected by BAs (**Figure** **2**A,B).

### 2.4 LCA Inhibited Platelet Integrin αIIbβ3 Activation, Granule Secretion, Clot Retraction, and Calcium Mobilization

**Figure 3:**
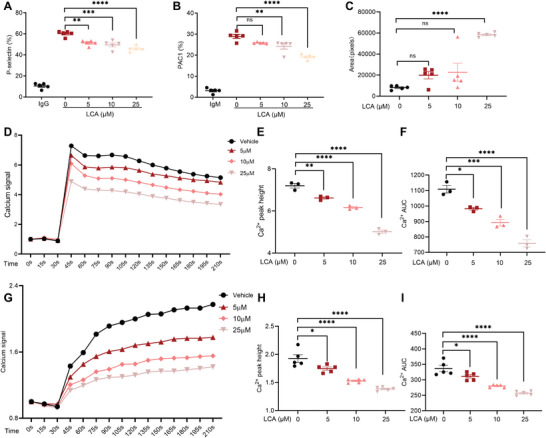
LCA inhibited platelet integrin αIIbβ3 activation, granule secretion, clot retraction, and calcium mobilization. A,B) Human washed platelets labelled with A) FITC‐conjugated P‐selectin or B) FITC‐conjugated PAC‐1 antibodies were incubated with LCA (5, 10, or 25 µm) or vehicle at 37 °C for 5 min and then stimulated with collagen; *n* = 5. C) Human washed platelets were incubated with LCA (5, 10, or 25 µm) or vehicle for 120 min in the presence of fibrinogen (400 µg mL^−1^) and 1 mm CaCl_2_, and clot retraction was initiated by thrombin; *n* = 5. D–F) Ca^2+^ mobilization induced by thrombin was demonstrated by Fluo‐3 fluorescence signal, which was monitored over time using flow cytometry in human washed platelets pretreated with LCA (0, 5, 10, or 25 µm). D) Representative curve of the Ca^2+^ fluorescence signal. E) The maximum Ca^2+^ signal and F) AUC of Fluo‐3 fluorescence signal was quantified; *n* = 3. G–I) Ca^2+^ mobilization induced by collagen was demonstrated by Fluo‐3 fluorescence signal, which was monitored over time using flow cytometry in human‐washed platelets pretreated with LCA (0, 5, 10, or 25 µm). G) Representative curve of the Ca^2+^ fluorescence signal. H) The maximum Ca^2+^ signal and I) AUC of Fluo‐3 fluorescence signal were quantified; *n* = 5. Data are presented as the mean ± standard error of the mean; one‐way ANOVA; ^*^ *p* < 0.05, ^**^ *p* < 0.01, ^***^ *p* < 0.001, ^****^ *p* < 0.0001, NS indicates no significance.

When platelets are activated, membrane receptors induce intracellular signals to trigger the inside‐out signaling process, which promotes the activation of integrin αIIbβ3 and secretion of alpha and dense granules. Subsequently, the activated αIIbβ3 binds to extracellular ligands and triggers outside‐in signaling, causing further activation of platelets.^[^ ^31^ ^]^ PAC‐1 binding can represent the activation of integrin αIIbβ3, and p‐selectin expression can determine the extent of α granules released from platelets. LCA considerably inhibited PAC‐1 binding and p‐selectin exposure in collagen‐treated human platelets (**Figure** **3**A,B). In addition, we also found that BAs could inhibit PAC‐1 binding and p‐selectin exposure in collagen‐treated human platelet‐rich plasma (PRP) (Figure S3A,B, Supporting Information). The inhibitory effect of secondary BAs was stronger than that of primary BAs, and the inhibitory effect of LCA is the most obvious.

Consistent with the effects of LCA on the inhibition of platelet spreading, LCA alleviated the contraction of clots in a human platelet suspension, which is a late outside‐in signaling event that occurs during platelet activation (Figure 3C).^[^ ^32^ ^]^ Intracellular calcium signal transduction plays an essential role in platelet activation.^[^ ^33^ ^]^ Under the stimulation of several agonists, intracellular calcium concentration increases to promote platelet activation.^[^ ^34^ ^]^ We assessed the effect of LCA on Ca^2+^ signaling in human platelets via flow cytometry. Intracellular Ca^2+^ concentration increased rapidly after platelets were stimulated by thrombin. While under the treatment of LCA, the peak Ca^2+^ concentration (peak height) and total influx (area under the curve; AUC) decreased. The inhibitory effect became more obvious as LCA concentration increased (Figure 3D–F). Thus, LCA inhibited calcium mobilization during platelet activation. Similarly, we examined the effect of LCA on calcium mobilization in human platelets stimulated by collagen, which also showed an inhibitory effect (Figure 3G–I).

### 2.5 LCA Suppressed Arterial Thrombosis and Did Not Affect Venous Thrombosis and Hemostasis

**Figure 4:**
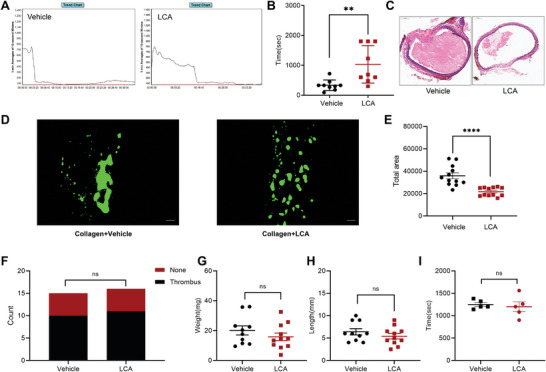
LCA suppressed arterial thrombosis, but had no effect on venous thrombosis and hemostasis. A,B) Thrombus formation in the carotid artery was induced by 10% FeCl_3_. The occlusion time of LCA (5 mg k^−1^g) group was compared with that of the vehicle group. C) Representative histological images of thrombi with H&E staining are shown; Scale bar = 50 µm. D,E) The human platelets labeled with Cell Trace Calcein Green were incubated with vehicle or LCA (25 µm) for 5 min and then perfused in Bioflux plate channels for 10 min. The representative images of platelets aggregated on the collagen surface are shown; Scale bar = 50 µm; N = 12. (F–H) DVT induced by stenosis of inferior vena cava was measured between mice treated with vehicle and those treated with LCA (5 mg k^−1^g) (Vehicle, 10 out of 15; LCA 11 out of 16). Furthermore, G) weight and H) length of thrombi were compared between the two groups (Weight: LCA 15.85 ± 8.430 mg, *n* = 11; Vehicle: 20.13 ± 9.594 mg, *n* = 10; *p*  = 0.2896) (Length: LCA 5.409 ± 2.01 mm, *n* = 11; Vehicle 6.4 ± 2.183 mm, *n* = 10; *p* = 0.2923). I) Tail bleeding assays were performed for the vehicle or LCA (5 mg k^−1^g)‐treated groups; *n* = 5. Data are presented as the mean ± standard error of mean; Unpaired *t*‐test, one‐way ANOVA; ^*^ *p* < 0.05, ^**^ *p* < 0.01, ^***^ *p* < 0.001, ^****^ *p* < 0.0001, NS indicates no significance.

We used the FeCl_3_ injury‐induced carotid thrombosis model to evaluate the effects of LCA on arterial thrombosis in vivo. Compared with vehicle‐treated mice, the occlusion time of the LCA‐treated mice was prolonged, indicating that LCA inhibited arterial thrombosis in vivo (**Figure** **4**A,B). Histological analysis of the thrombus by H&E staining showed reduced vascular obstruction after 8 min in LCA (5 mg k^−1^g)‐treated mice (Figure 4C). In addition, to explore the effect of primary bile acid (CA) on the formation of carotid artery thrombosis in mice, we treated mice with CA (5 mg k^−1^g) and found that there was no significant difference in the formation time of carotid artery thrombosis between the mice treated with CA and the control group (Figure S4A,B, Supporting Information). In vivo, circulation shear stress caused by blood flow is an important factor in thrombosis.^[^ ^33^ ^]^ To further elucidate the inhibitory effect of LCA on thrombosis in vivo, a microfluidic system was used to mediate human platelet adhesion to collagen matrix under high shear stress, similar to conditions in vascular injury or stenosis. LCA significantly reduced platelet adhesion to collagen matrix under arterial shear (Figure 4D,E). Venous thrombosis differs from arterial thrombosis, which is usually caused by blood flow stasis.^[^ ^35^ ^]^ The inferior vena cava ligation model revealed that the venous thrombus length and weight did not significantly differ between LCA (5 mg k^−1^g)‐treated mice and vehicle‐treated mice (Figure 4F–H). Therefore, LCA treatment probably did not affect the development of venous thrombosis. In addition, we verified the role of LCA in hemostasis in a mouse tail bleeding experiment. The bleeding time was comparable between LCA‐treated mice and vehicle‐treated mice (Figure 4I). Therefore, LCA might have had no effect on hemostasis.

### 2.6 NCK1 is Involved in Platelet Activation

**Figure 5:**
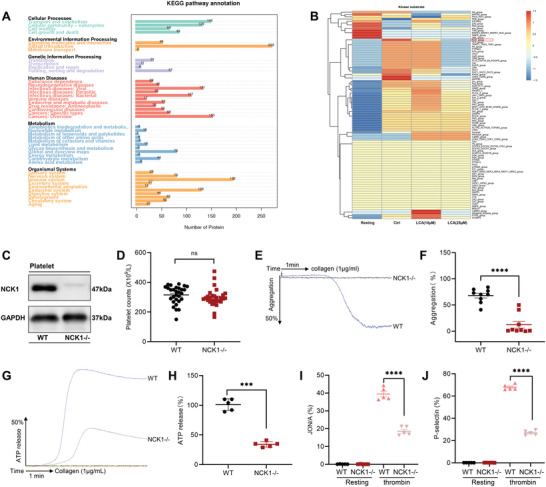
NCK1 was involved in platelet activation. A) Kyoto Encyclopedia of Genes and Genomes pathway enrichment of human platelets treated with LCA was analyzed. B) Phosphoproteomic analysis of the impact of LCA (0, 10, or 25 µm) treatment on the kinase‐substrate relationships in platelets; *n* = 3 C) The platelets of WT and NCK1−/− mice were extracted to determine the expression level of NCK1. D) Platelet counts in peripheral blood of WT and NCK1−/− mice. E,F) Platelet aggregation in WT and NCK1−/− mice stimulated with collagen (1 µg mL^−1^); *n* = 9. G,H) Collagen‐stimulated platelet ATP release in WT and NCK1−/− mice; *n* = 5. I) JON/A binding and J) P‐selectin exposure of WT and NCK1−/− mice platelets in response to 0.08 U/mL thrombin; *n* = 5. Data are presented as the mean ± standard error of mean; Unpaired *t*‐test, one‐way ANOVA; ^*^ *p* < 0.05, ^**^ *p* < 0.01, ^***^ *p* < 0.001, ^****^ *p* < 0.0001, NS indicates no significance.

To elucidate the mechanisms of LCA‐mediated effects on platelet activation, the phosphoproteome of LCA‐treated human platelets was analyzed. A total of 1 536 modified proteins and 5 619 modified sites were identified in the experimental group. Kyoto Encyclopedia of Genes and Genomes pathway enrichment analysis of modified proteins revealed multiple enriched pathways, including MAPK, calcium/calmodulin‐dependent protein kinase II delta, and others (**Figure** **5**A). In addition, prediction analysis of phosphorylated kinases for the identified modification sites showed that NCK was significantly reduced after LCA treatment compared with those in the vehicle‐treated group (Figure 5B). Therefore, NCK may be involved in the inhibitory effect of LCA in platelet activation. In our study, we focused on the role of NCK1 in platelet activation, and NCK1−/− mice were used in subsequent studies (Figure 5C). The whole blood of mice was obtained and then platelets were counted using a blood cell counter. NCK1 deficiency did not affect the platelet count in peripheral blood (Figure 5D), indicating that NCK1 deficiency did not affect megakaryocyte maturation and platelet production. In addition, we found that NCK1 expression was not affected by LCA and NCK1 deficiency did not affect GPVI, Integrin β3, and GPIb expression (Figure S5A,B, Supporting Information). For collagen‐stimulated platelet aggregation, wild‐type (WT) platelet aggregation was greater than that of NCK1−/− platelets (Figure 5E,F). Moreover, NCK1 deficiency decreased ATP secretion (Figure 5G–H), JON/A binding, and P‐selectin exposure of platelets (Figure 5I,J), suggesting that NCK1 may regulate platelet activation by affecting granular secretion and the inside‐out and outside‐in signaling processes. To further understand the mechanism underlying this effect, we collected the aggregation products of mouse platelets for protein immunoblotting. The results showed that NCK1 deficiency inhibited the phosphorylation of SYK, ERK1/2, and AKT in platelets, which was consistent with the results of human platelet (Figure S5C,D, Supporting Information).

### 2.7 NCK1 Deficiency Impaired Arterial Thrombosis and Did Not Affect Venous Thrombosis and Hemostasis

**Figure 6:**
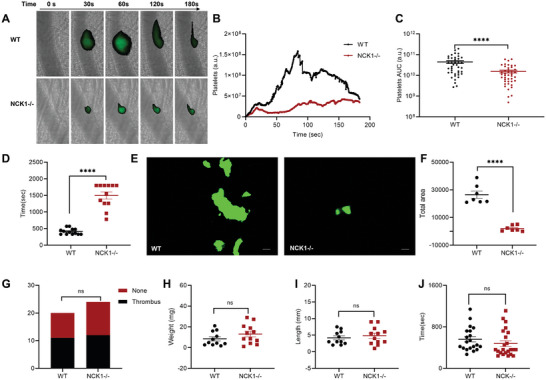
NCK1 deficiency impaired arterial thrombosis but did not affect venous thrombosis and hemostasis. A) WT and NCK1−/− mice were subjected to surgery to expose the cremaster arterioles, followed by laser stimulation to induce thrombus. Representative images of platelet accumulation at the injury site at different time points are shown. B) The medium fluorescence intensity for platelets is plotted over time for WT and NCK1−/− groups. C) The AUC for platelets from all captures was analyzed. D) Thrombus formation in the carotid artery was induced by 10% FeCl_3_. The occlusion time was 413.1 ± 90.13 s in WT mice (*n* = 13), 1498 ± 368.2 s in NCK1−/− mice (*n* = 12). E,F) The platelets of WT and NCK1−/− mice labeled with Cell Trace Calcein Green were perfused in Bioflux plate channels for 10 min, and then the images were obtained. The representative images of platelets aggregated on the collagen surface are shown; Scale bar = 50 µm; *n* = 7. G–I) DVT induced by stenosis of inferior vena cava was measured in WT and NCK1−/− mice. H) Weight: WT 8.445 ± 6.773 mg, *n* = 11, NCK1−/− 13.06 ± 9.337 mg, *n* = 12; *p* = 0.193. I) Length: WT 4.182 ± 2.016 mm, *n* = 11, NCK1−/− 4.792 ± 2.65 mm, *n* = 12; *p* = 0.5441. J) Tail bleeding assay was performed for WT and NCK1−/− mice. Data are presented as the mean ± standard error of mean; unpaired *t*‐test, one‐way ANOVA; ^*^ *p* < 0.05, ^**^ *p* < 0.01, ^***^ *p* < 0.001, ^****^ *p* < 0.0001, NS indicates no significance.

For evaluating arterial thrombosis, we used a laser‐induced arterial injury model, in which platelets were labeled with DyLight488‐conjugated anti‐CD42c antibodies. The cremaster arteriole of NCK1−/− and WT mice were monitored under an intravital microscope. Laser stimulation was used to induce living arteriole thrombus formation, and we found that platelet accumulation in NCK1−/− mice was significantly decreased compared with that in WT mice (**Figure** **6**A–C). Furthermore, NCK1 deficiency prolonged the occlusion time in the FeCl_3_‐induced carotid thrombosis model (Figure 6D) and inhibited platelet‐collagen adhesion under simulated arterial shear stress in vitro (Figure 6E,F). For evaluating venous thrombosis, we established an inferior vena cava stenosis model and found that thrombus length and weight did not significantly differ between NCK1−/− and WT mice (Figure 6G–I). In addition, mouse tail bleeding experiments showed that bleeding time was comparable between WT and NCK1−/− mice (Figure 6J).

### 2.8 LCA Inhibited Syntaxin‐11 Phosphorylation in Platelets, and Syntaxin‐11 Phosphorylation on S80 and S81 was Involved in αIIbβ3 Signal Transduction

**Figure 7:**
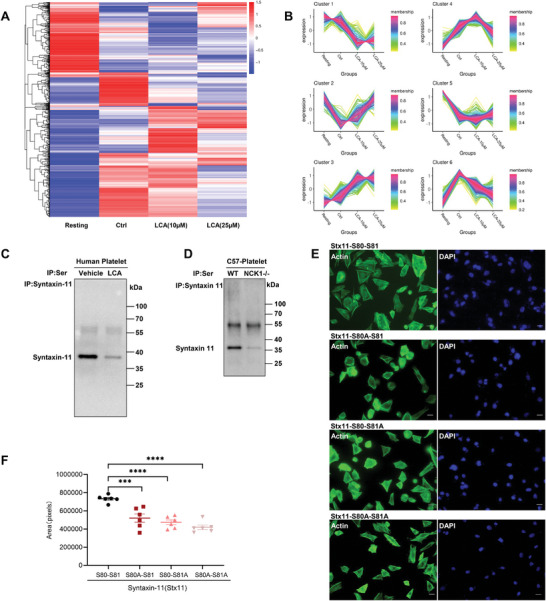
LCA inhibited syntaxin‐11 phosphorylation in platelets, and syntaxin‐11 phosphorylation on S80 and S81 is involved in αIIbβ3 signal transduction. A,B) Phosphoproteomic analysis in platelets treated with vehicle or LCA (0, 10, or 25 µm). According to the screening criteria, the A) heat map and B) clustering diagram of differential protein (union) expression levels were depicted; *n* = 3 C) Serine phosphorylation level of syntaxin‐11 was inhibited by LCA in human platelets. D) Serine phosphorylation level of syntaxin‐11 from WT and NCK1−/− mice platelets. E,F) Representative immunofluorescence actin staining images and DAPI images of αIIbβ3‐CHO cells transfected with Stx11‐flag, Stx11‐S80A‐flag, Stx11‐S81A‐flag, or Stx11‐S80A‐S81A‐flag plasmids spreading on immobilized fibrinogen; *n* = 6; Data are presented as the mean ± standard error of mean; Unpaired *t*‐test, one‐way ANOVA; ^*^ *p* < 0.05, ^**^ *p* < 0.01, ^***^ *p* < 0.001, ^****^ *p* < 0.0001, NS indicates no significance.

Phosphorylation of protein serine residues (Ser) represents an important posttranslational modification that regulates protein function.^[^ ^36^ ^]^ In our study, phosphorylation site analysis showed that phosphorylation varied between proteins (**Figure** **7**A). Among the proteins, syntaxin‐11 phosphorylation was inhibited by LCA in a concentration‐dependent manner (Figure 7B). To verify this observation, we performed co‐immunoprecipitation analysis of human platelets, showing that pre‐incubation with LCA significantly inhibited Ser phosphorylation of syntaxin‐11 (Figure 7C). Moreover, the level of Ser phosphorylation of syntaxin‐11 in NCK1−/− platelets decreased compared with that in WT platelets (Figure 7D). Previous findings indicated that syntaxin plays a critical role in regulating platelet secretion and actin cytoskeleton development.^[^ ^37^ ^]^ The bidirectional signaling mediated by integrin αIIbβ3 plays an essential role in platelet deformation and retraction.^[^ ^32^, ^38^ ^]^ To elucidate the role of syntaxin‐11 in platelet signal transduction, we used αIIbβ3‐expressing CHO cells, which represent an important functional cell line for studying integrin αIIbβ3 signaling in platelets.^[^ ^32^ ^]^ LCA suppressed αIIbβ3‐CHO spreading on the plates covered with fibrinogen (Figure S6A,B, Supporting Information). In addition, phosphorylation site analysis of platelets identified syntaxin‐11 phosphorylation at S80 and 81. To evaluate the importance of the phosphorylation site of syntaxin‐11, we constructed plasmids related to protein mutations at specific amino acid sites and performed spreading experiments. The fluorescent spreading area of αIIbβ3‐CHO cells transfected with the single or double mutation vector of the syntaxin‐11‐S80(A)/S81(A) was significantly decreased compared with that of αIIbβ3‐CHO cells transfected with the syntaxin‐11‐flag plasmid (Figure 7E,F). These results suggest that S80 and S81 are important phosphorylation sites on syntaxin‐11 and play an essential role in integrin αIIbβ3 signal transduction.

### 2.9 LCA was Correlated with Atherosclerosis

**Figure 8:**
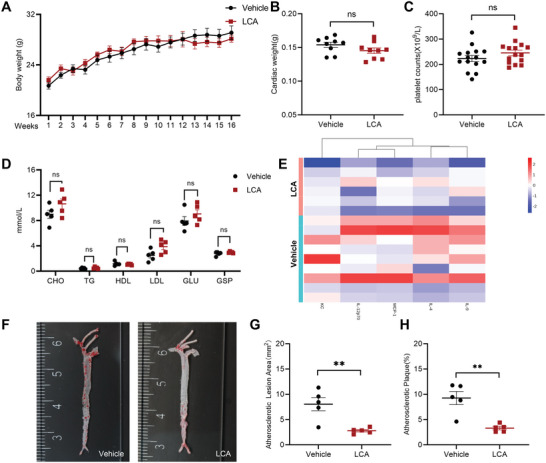
LCA reduced atherosclerotic plaque formation in mice. A) Body weight changes in response to HFD feeding in LCA‐treated mice and vehicle‐treated mice. The weight of each group was recorded weekly for 16 weeks. B) The cardiac weight of mice and the C) platelet counts of blood were recorded after 16 weeks. D) CHO, TG, HDL, LDL, GLU, and GSP levels were measured. E) Heat map representation of cytokine concentration in serum samples was analyzed using Quantibody mouse cytokine array kit. The screening criteria were: *p* < 0.05 after correction, difference factor greater than 1.2, and less than 0.83. F–H) Representative images of total aortic lesions stained with oil red O. G) Total aortic atherosclerotic plaque area and H) proportion of atherosclerotic plaque area within the whole aortic surface area. Data are presented as the mean ± standard error of mean; Unpaired *t*‐test, one‐way ANOVA; ^*^ *p* < 0.05, ^**^ *p* < 0.01, ^***^ *p* < 0.001, ^****^ *p* < 0.0001, NS indicates no significance.

To evaluate the role of LCA in AS, we established an AS model by feeding mice with HFD. The body weight of the mice was measured weekly, and the mouse tissues were obtained after 16 weeks. Body weight was comparable between LCA‐treated and vehicle‐treated mice, (**Figure** **8**A) and no significant changes in heart weight (Figure 8B), peripheral blood platelet count (Figure 8C), and plasma lipid and blood glucose indexes (plasma cholesterol, triglycerides, high‐density lipoprotein, low‐density lipoprotein, glucose, glycated serum protein) were observed (Figure 8D). However, the plasma levels of inflammatory factors such as interleukin 4 (IL‐4), IL‐9, IL‐12p70, keratinocyte chemoattractant (KC), and monocyte chemoattractant protein‐1 (MCP‐1) were decreased in LCA‐treated mice (Figure 8E). Atherosclerotic lesion formation was assessed in the aorta and carotid artery. Enface analysis of AS in the artery was assessed via Oil red O staining and quantified by calculating the lesion area and the proportion of lesion area in the total aortic surface area. LCA‐treated mice had significantly reduced aortic plaque burden (Figure 8F–H). Moreover, LCA‐treated mice showed significant alleviation of plaque burden in the carotid artery (Figure S7A,B, Supporting Information). To evaluate the characteristics of atherosclerotic plaques in this model, macrophage staining of plaques was performed, and the area of macrophages in the carotid artery of LCA‐treated mice was found to be significantly reduced (Figure S7C, Supporting Information).

## 3 Discussion

BAs are products of cholesterol metabolism and exert biological effects in various organs and tissues of the human body. In addition to their physiological effects, BAs play an essential role in the development of thrombotic diseases. Previous studies have shown that in rats, BAs can inhibit platelet aggregation, and the use of 1.25% rabbit bile can inhibit ADP‐induced platelet aggregation.^[^ ^25^, ^39^ ^]^ This indicates that BAs have a negative regulatory effect on platelet activation. However, the role of different BAs in platelet activation and thrombotic pathogenesis remains unknown. Our study demonstrated that BAs inhibited platelet activation, a central feature of arterial thrombotic diseases, suggesting that the signaling pathways associated with BAs may serve as therapeutic targets in arterial thrombosis.

According to previous studies, two primary BAs are found in humans: CA and CDCA. In the intestine, CA is converted into DCA and CDCA is converted into LCA.^[^ ^40^, ^41^ ^]^ Therefore, in our study, we mainly explored the regulatory effects of these four BAs on platelet function. Among these four BAs, we found that secondary BAs play a more significant role in regulating platelet activation than that of primary BAs. Under the stimulation of different agonists, the inhibitory effect of BAs on platelet aggregation differs, indicating that the signal transduction of BAs inhibiting platelet activity is not applicable to all agonists. For example, under the same concentration of bile acid, DCA, the CA can inhibit the platelet activation induced by collagen, and does not affect the platelet activation induced by thrombin. Moreover, we found that the inhibitory effect of these four BAs on platelet spreading and release function differed. Our results showed that secondary bile acids had a stronger inhibitory effect on platelet function than primary bile acids, and LCA had the most significant inhibitory effect. Therefore, we further explored the role of LCA in arterial and venous thrombosis in mice, and found that LCA could inhibit the formation of carotid artery thrombosis induced by ferric chloride in mice. However, LCA has no effect on venous thrombosis, this may be due to the different pathophysiological mechanisms of arterial and venous thrombosis. That is, the thrombus formed by venous thrombosis is rich in fibrin and red blood cells, while the thrombus formed by arterial thrombosis is rich in platelets.^[^ ^42^ ^]^ In addition, we demonstrated that LCA had no effect on the tail bleeding time of mice at the experimental concentration, indicating that LCA has no significant regulatory effect on the coagulation system, further demonstrating that inhibition of platelet signaling by LCA is an effective and safe antithrombotic strategy.

To further study the inhibition of platelet activation, we collected platelet aggregates after collagen stimulation and performed western blotting. We found that the inhibitory effects of these four BAs on the downstream signaling pathways of platelet GPVI differed. The phosphorylation levels of signaling molecules, including SYK, AKT, and ERK differed, which also explained the differences in platelet inhibition levels. Notably, we found no difference in phosphorylation levels of P38 signaling molecules, suggesting that the downstream effects of BAs on platelet activation are not mediated by P38. Phosphorylation is an important process that mediates cell mobilization and emergency response, which promotes physiological and pathological processes by altering the structure of proteins leading to changes in activity or function.^[^ ^43^ ^]^ Further, protein phosphorylation is vital for maintaining the functions of the platelet.^[^ ^44^ ^]^ Thus, we used phosphorylated proteomics to analyze the mechanism underlying LCA‐mediated inhibition of platelets stimulated by collagen. Based on the analysis of the results, we found that syntaxin‐11 may play a critical role in LCA‐mediated inhibition of platelet activity.

Syntaxin‐11 is an atypical member of the family of soluble N‐ethylmaleimide‐sensitive factor attachment protein receptors (SNAREs); it does not contain a traditional transmembrane domain. SNAREs, which are membrane‐trafficking proteins, can regulate trafficking and membrane fusion between organelles or the cell surface.^[^ ^45^, ^46^, ^47^ ^]^ Syntaxin‐11 is mainly expressed in the thymus, spleen, and lymph nodes of the immune system.^[^ ^48^ ^]^ Recent studies have shown that syntaxin‐11 is expressed in platelets and is the most abundant subtype in platelets.^[^ ^49^ ^]^ In the absence of syntaxin‐11, platelets show impaired secretion of dense and α‐granules, indicating that syntaxin‐11 participates in platelet secretion.^[^ ^50^ ^]^ Ye claimed that syntaxin‐11 forms a complex with SNAP23‐vesicle‐associated membrane protein 8 (VAMP8) which is involved in the exocytosis of platelet alpha and dense granules, further establishing that it is a key element in the mechanism underlying platelet secretion.^[^ ^49^ ^]^ Moreover, VAMP‐8 and syntaxin‐2 are associated with actin cytoskeleton development, and platelet SNAREs are associated with platelet cytoskeleton reorganization, which can ultimately affect a change in platelet shape and activation.^[^ ^37^ ^]^ In the present study, syntaxin‐11 phosphorylation was found to be decreased in LCA‐treated platelets. Phosphorylation of S80 and S81 was found to play a critical role in αIIbβ3 activation and platelet activation, demonstrating the functional mechanism of phosphorylation of specific sites of syntaxin‐11.

The NCK (noncatalytic region of tyrosine kinase) family of adaptor proteins includes NCK1 and NCK2 comprising SH2/SH3 domains, which can mediate protein‐protein interactions. Nck1 and Nck2 share 68% amino‐acid identity1.^[^ ^51^, ^52^ ^]^ Interactions between Nck's SH2 domain and tyrosine phosphorylated proteins recruits NCK to sites of active cell signaling, where it brings in other signaling mediators through interactions between its SH3 domains and proline‐rich sequences in target proteins, ultimately mediating protein–protein interactions.^[^ ^53^ ^]^ NCK signaling can induce phosphorylation of intracellular signaling proteins to mediate cytoskeletal remodeling and membrane protrusions.^[^ ^54^ ^]^ Mabruka Alfaidi's work provides the evidence that Nck1 but not the highly similar Nck2 plays a distinct role in disturbed flow‐induced vascular permeability, highlighting a clearly nonredundant functional difference between Nck1 and Nck2.^[^ ^53^ ^]^ In addition, further studies have suggested that NCK1, but not NCK2 plays a distinct role in atherogenic inflammation and plaque formation.^[^ ^55^ ^]^ Kinase analysis in our study showed that NCK may play an essential role in collagen‐stimulated platelet activation and is affected by LCA. Meanwhile, it has been reported that the down‐regulation of Nck1 in lens epithelial cells impairs PDGFR‐induced phosphorylation of intracellular signaling proteins, including Erk1/2 and Akt,^[^ ^56^ ^]^ which is consistent with the downstream signaling pathway that BAs affect platelet activation in our study. Given the non‐redundant roles of NCK1 and NCK2 in inflammation and atherosclerosis, we hypothesized that NCK1 might play an important role in platelet activation and thrombosis. Therefore, we constructed *Nck1*‐knockout mice and found that when NCK1 was deficient, platelet aggregation, granule release, adhesion, and other functions were suppressed, and laser‐induced arteriolar thrombosis was also reduced; however, it had no effect on coagulation, indicating that NCK1 is an important target for regulating platelet activation and thrombosis.

AS is the primary cause of arteriosclerotic cardiovascular disease.^[^ ^57^ ^]^ Several studies have shown that BAs play an essential role in the initiation and development of AS and are associated with AS risk factors, such as inflammation and oxidative stress.^[^ ^58^, ^59^ ^]^ The major BA molecular mediators (FXR and TGR5) exhibit anti‐atherosclerotic activities.^[^ ^21^, ^24^ ^]^ Other multiple BA‐responsive nuclear receptors exert anti‐atherosclerotic or pro‐atherosclerotic effects.^[^ ^60^, ^61^ ^]^ The excretion rate of fecal BAs in patients with coronary artery disease (CAD) is lower than that in healthy individuals, suggesting that BA excretion has a regulatory effect on the occurrence and development of CAD.^[^ ^62^, ^63^ ^]^ Recently, Li's study showed that patients with CAD had lower serum TBA levels than those without CAD,^[^ ^64^ ^]^ indicating that serum bile acids may be related to the occurrence of atherosclerosis. In the present study, to further investigate the effect of LCA on AS, a diet‐induced spontaneous AS model was established which showed that LCA could reduce the release of inflammatory factors and the formation of atherosclerotic plaques in mice, indicating that LCA is a protective factor for thrombosis and AS.

In general, we demonstrated that CA, DCA, CDCA, and LCA inhibited platelet activation, and LCA had the most obvious inhibitory effect. In addition, LCA inhibited carotid thrombosis and did not affect venous thrombosis and hemostasis in vivo. Furthermore, our results provide new insights into the relationship between platelet NCK1, platelet activation, and arterial thrombosis, and demonstrate the role of phosphorylation of sytanxin‐11 in these processes. Moreover, we found that additional LCA supplementation attenuated atherosclerotic plaque development and reduced inflammation in mice, which extends the current understanding of atherosclerotic cardiovascular diseases in response to BAs. Taken together, these findings provide important insights for exploring bile acids and their related mechanisms in the treatment of AS and thrombosis.

## 4 Experimental Section

### Human Studies and Informed Consent Statement

The study was approved by the Medical Ethics Committee, Union Hospital, Tongji Medical College, Huazhong University of Science and Technology (Approval no. 2020‐0245) and was conducted in accordance with the principles of the Declaration of Helsinki. Informed consents were obtained from all participants.

### Animal Studies

*Nck1* knockout (NCK1–/–) mice were purchased from Model Organisms Centre (Shanghai, China). Apolipoprotein E‐knockout (ApoE–/–) mice of C57BL/6J background were obtained from GemPharmatech (Nanjing, China). Animal experiments and surgical procedures were performed according to protocols approved by the institutional guidelines at the Institutional Animal Care and Use Committee at Tongji Medical College, Huazhong University of Science and Technology, following the ARRIVE guidelines. (Approval no. 2020‐S2324).

### Platelet Preparation and Functional Experiments

Platelets were prepared by gradient centrifugation, as described previously.^[^ ^65^ ^]^ To determine the function of BAs in platelet aggregation, 250 µL of 300 × 10^9^ platelets/L were incubated with LCA, CDCA, DCA, or CA for 5 min at 37 °C. Platelet aggregation was stimulated using agonists, including collagen (1 µg mL^−1^), thrombin (0.08 U mL^−1^), ADP (2.5 µm), AA (0.5 mm), and U46619 (0.12 µg mL^−1^). CHRONO‐LUME kit was used to detect ATP release. Platelet calcium signaling was analyzed using Fluo‐3AM. Platelet retraction assay and platelet spreading experiments were performed as described previously.^[^ ^65^ ^]^ Mouse platelet JON/A binding (PE‐conjugated JON/A antibody (Biolegend, San Diego, CA, USA)), human platelet PAC1 binding (fluorescein isothiocyanate [FITC]‐conjugated PAC1 antibody (Biolegend, San Diego, CA, USA)), phosphatidylserine exposure (FITC‐Annexin‐V (Biolegend, San Diego, CA, USA)) and human/mouse P‐selectin exposure (FITC‐conjugated anti‐P‐selectin antibody (Biolegend, San Diego, CA, USA)) were analyzed using different channels in a flow cytometer (BD Biosciences, Franklin Lakes, NJ, USA).^[^ ^65^ ^]^

### Lactate Dehydrogenate (LDH) Assay

Cytotoxic assay kits were used to measure LDH levels in platelets. Human platelets (300 × 10^9^/L) were prepared and incubated with BAs or solvent at 37 °C for 5 min. The supernatant (120 µL) was collected by centrifugation and transferred to a 96‐well plate. The prepared detection buffer (60 µL) was added to each well and incubated at room temperature for 30 min, away from light. Then the absorbance was detected at 490 nm.

### Flow Chamber Experiments

Bioflux plate channels (Fluxion Biosciences Inc., Oakland, CA USA) were coated with collagen (100 µg mL^−1^) and then placed overnight at 4 °C. Human or mouse platelets were stained with Cell Trace Calcein Green (Invitrogen, Waltham, MA, USA) at 37 °C for 30 min. The platelets were labeled with fluorescent dye using Bioflux plates at a shear rate of 20 dyn cm^−2^ for 10 min. Flowing platelets in the channel were observed under an epifluorescence microscope. Platelet adhesion was measured using a 10× long‐working‐distance objective.

### Thrombosis and Hemostasis Mouse Models

A laser‐induced mouse small‐artery thrombosis model was established as previously described.^[^ ^66^ ^]^ The mouse carotid artery thrombosis model was induced by FeCl_3_ after which blood flow monitoring was performed. Occlusion time was defined as the first time point when stable flow interruption was achieved or when 30 min had passed. The changes in blood flow over time were recorded. 8 min later, the carotid artery was harvested, fixed with paraformaldehyde, embedded, and sectioned for hematoxylin‐eosin staining. A model of deep venous thrombosis (DVT) induced by stenosis of the inferior vena cava was established as described previously. Mice were anesthetized with 3% isoflurane. The inferior vena cava (IVC) was surgically exposed and separated from the surrounding tissue. The collateral branches of the inferior vena cava were ligated. The IVC was then partially ligated using 8‐0 polypropylene sutures, resulting in IVC stenosis and significant flow restriction. Venous thrombosis was observed 48 h after the operation.^[^ ^65^ ^]^ Tail bleeding test was performed as previously described.^[^ ^65^ ^]^ The mice involved in the above experiments were 8–10 weeks old, and the age of each experimental group was kept consistent.

### Plasmid Constructs and Transfection

A plasmid related to *Stx11* was constructed by Shanghai Genechem (Shanghai, China). *Stx‐11* cDNA was cloned into a p‐CMV‐flag vector. Based on the p‐CMV‐flag‐Stx‐11 plasmid vector, the Ser80 or 81 site of Stx‐11 was mutated to alanine to prevent phosphorylation. For transfection, αIIbβ3‐CHO cells were obtained from the research group of Prof Jun‐ling Liu (Shanghai Jiao Tong University, Shanghai, China) and transfection was performed using Lipofectamine 3000 (Invitrogen, Carlsbad, CA, USA). αIIbβ3‐CHO cells were transfected with a constructed plasmid (flag‐tagged Stx‐11, flag‐tagged Stx‐11‐ser80A, flag‐tagged Stx‐11‐ser81A, and flag‐tagged Stx‐11‐ser80A‐ser81A) according to the manufacturer's instructions. After incubation at 37 °C for 24 h, the medium was replaced with fresh normal medium following incubation for an additional 24 h. The transfected αIIbβ3‐CHO cells were collected for spreading on fibrinogen.

### Western Blotting and Co‐Immunoprecipitation

Platelet aggregation reaction products were prepared for western blotting by boiling with loading buffer and then separated via gel electrophoresis. Phosphorylated‐spleen tyrosine kinase, p‐p38‐mitogen‐activated protein kinase (MAPK), p‐extracellular signal‐regulated kinase 1/2, and p‐protein kinase B (Cell Signaling Technology, Danvers, MA, USA) were used as primary antibodies, and the membranes were then incubated with secondary antibodies. Bands were quantified using ImageJ analysis (National Institutes of Health, Bethesda, MD, USA). For co‐immunoprecipitation assays, platelet aggregation reaction products were prepared. The immunoprecipitation lysates were incubated with the specified antibody or control IgG and placed on a rotary instrument overnight at 4 °C. Subsequently, 30 µL protein A + G agarose was added and continued to rotate at 4 °C for 2 h. The beads were harvested via centrifugation and washed five times with phosphate‐buffered saline (PBS). After boiling with loading buffer, immunoprecipitation samples were analyzed via western blotting.

### AS Model and Assessment of Atherosclerotic Lesions

Four‐week‐old ApoE–/– mice were fed with a normal diet for one week, followed by 16 weeks of high‐fat diet (HFD) feeding. Caudal intravenous injection of LCA or vehicle was performed once every 2 d, and body weight was monitored weekly. Finally, the mice were anesthetized (pentobarbital sodium: 80 mg k^−1^g) to obtain venous blood, heart, and whole aortic tissues. After the liquid was adsorbed by filter paper, the hearts were weighed and recorded. Platelet count was determined by a complete blood cell counter. The venous blood was centrifuged to obtain the serum, and the cytokines in the samples were detected using the Quantibody mouse cytokine array kit (QAM‐CYT‐1; RayBiotech, Peachtree Corners, GA, USA) according to the manufacturer's instructions, and the serum levels of CHO, TG, HDL‐C, LDL‐C, GLU, GSP were detected. Atherosclerotic plaques in mice were evaluated using the whole aorta and by quantifying plaque area and its proportion among the entire area of the aorta.^[^ ^67^ ^]^ In addition, hematoxylin and eosin (H&E) and immunofluorescence staining were performed to evaluate the extent of AS in the cross section of the carotid artery.^[^ ^68^ ^]^

### Statistical Analysis

All data were tested for significance using two‐tailed Student's *t*‐tests or one‐way analysis of variance (ANOVA). Data are presented as the mean ± standard error of the mean. Values of *p* < 0.05 were considered significant. GraphPad Prism software (GraphPad, San Diego, CA, USA) was used for statistical analysis.

## Conflict of Interest

The authors declare no conflict of interest.

## Supporting information

Supporting Information

## Data Availability

The data that support the findings of this study are available in the supplementary material of this article.
