# Supplementary material for: Mechanism of Bile Acid in Regulating Platelet Function and Thrombotic Diseases
Source: Adv Sci (Weinh). 2024 Jun 23;11(32):2401683. doi: 10.1002/advs.202401683 (PMC11348205; doi:10.1002/advs.202401683)
Supplement: Supplementary file 1 — Supporting Information [file ADVS-11-2401683-s001.pdf]

ADVANCED SCIENCE
Open Access

Supporting Information

for *Adv. Sci.*, DOI 10.1002/advs.202401683

Mechanism of Bile Acid in Regulating Platelet Function and Thrombotic Diseases

Xianghui Zhou, Xin Zhou, Zhao Zhang, Ruirui Zhu, Meng Lu, Keyu Lv, Chao Fang, Zhangyin Ming, Zhipeng Cheng and Yu Hu**

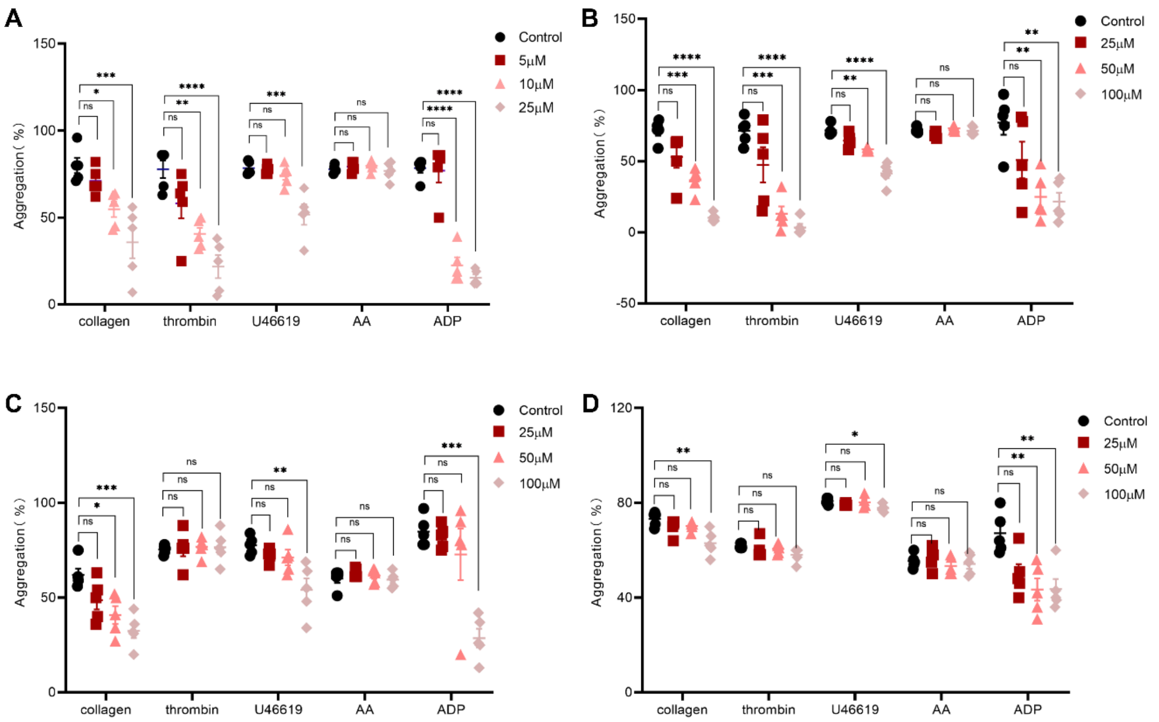

Supplemental Figure 1. BAs inhibited platelet aggregation.

(A–D) Washed human platelets were prepared for aggregation. The platelet suspension was pre-incubated with (A) LCA, (B) CDCA, (C) DCA, or (D) CA for 5 min, and then placed in an aggregator. After the baseline was stable, collagen (1 µg/mL), thrombin (0.08 U/mL), U46619 (0.12 µg/mL), and AA (0.5 mM) were added to induce platelet aggregation. PRP obtained from humans was pretreated with (A) LCA, (B) CDCA, (C) DCA, or (D) CA and stimulated with ADP (2.5 µM); N = 5. Three concentrations of every BAs were used as indicated. Data are presented as the mean ± standard error of mean; one-way ANOVA; *P < 0.05, **P < 0.01, ***P < 0.001, ****P < 0.0001, NS indicates no significance. PRP, platelet-rich plasma; LCA, lithocholic acid; CDCA chenodeoxycholic acid; DCA, deoxycholic acid; CA, cholic acid; BA, bile acid.

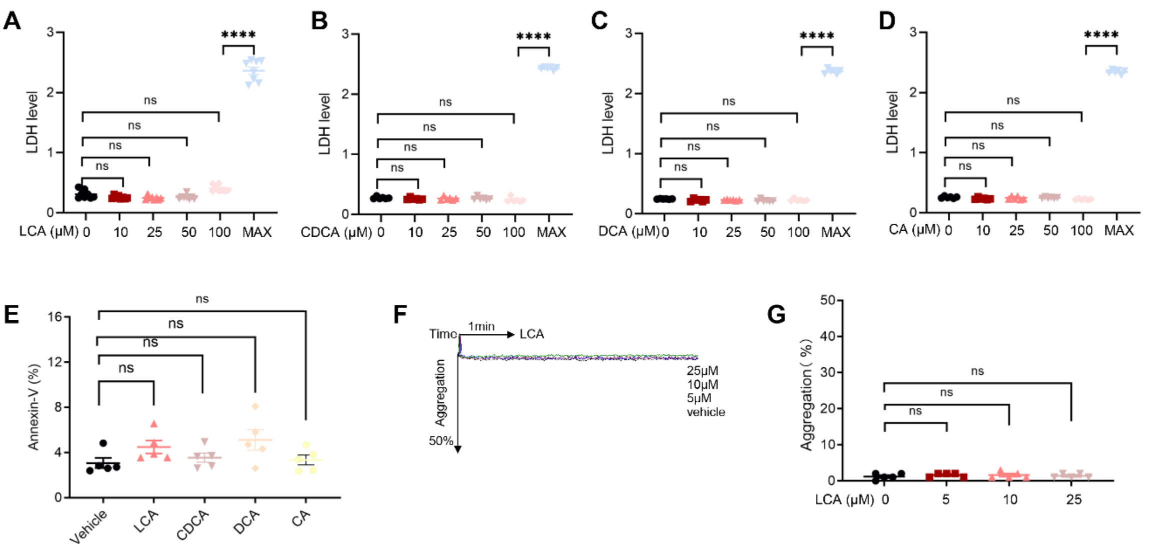

Supplemental Figure 2. The effect of BAs on LDH release and phosphatidylserine exposure.

(A-D) LDH was determined in washed platelets treated with BAs or vehicle, Max group is the addition of lysis buffer. (E) Resting state human washed platelets labelled with Annexin-V binding were incubated with Vehicle, LCA (25 μM), CDCA (25 μM), DCA (25 μM), CA (25 μM); N = 5. (F, G) Washed platelets from human were pretreated with 1 mM $CaCl_2$ for 5 min and stimulated with different doses of LCA; N = 5. Data are presented as the mean ± standard error of mean; one-way ANOVA; *P < 0.05, **P < 0.01, ***P < 0.001, ****P < 0.0001, NS indicates no significance.

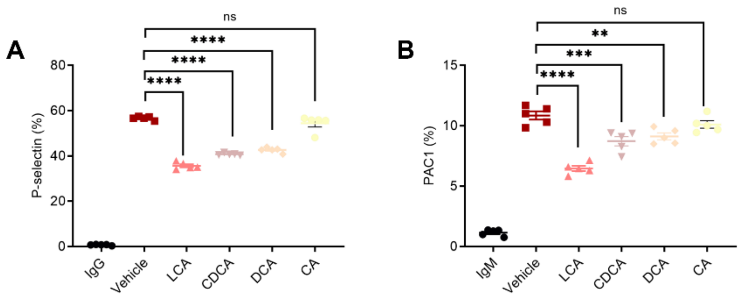

Supplemental Figure 3. The effect of BAs on P-selectin exposure and PAC-1 binding in platelet rich plasma (PRP).

(A, B) Human PRP labelled with (A) FITC-conjugated P-selectin or (B) FITC-conjugated PAC-1 antibodies were incubated with LCA (25 μM), CDCA (25 μM), DCA (25 μM), CA (25 μM) or vehicle at 37 °C for 5 min and then stimulated with collagen; N = 5. Data are presented as the mean ± standard error of mean; one-way ANOVA; *P < 0.05, **P < 0.01, ***P < 0.001, ****P < 0.0001, NS indicates no significance.

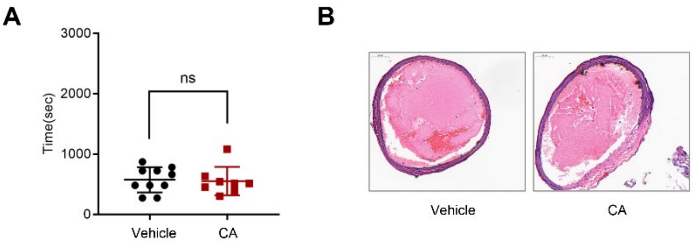

Supplemental Figure 4. The effect of CA on arterial thrombosis.
(A) Thrombus formation in the carotid artery was induced by 10% $FeCl_3$. The occlusion time of CA (5 mg/kg) group was compared with that of the vehicle group. (B) Representative histological images of thrombi with H&E staining are shown; Scale bar =50 μm. N= Data are presented as the mean ± standard error of mean; one-way ANOVA; *P < 0.05, **P < 0.01, ***P < 0.001, ****P < 0.0001, NS indicates no significance.

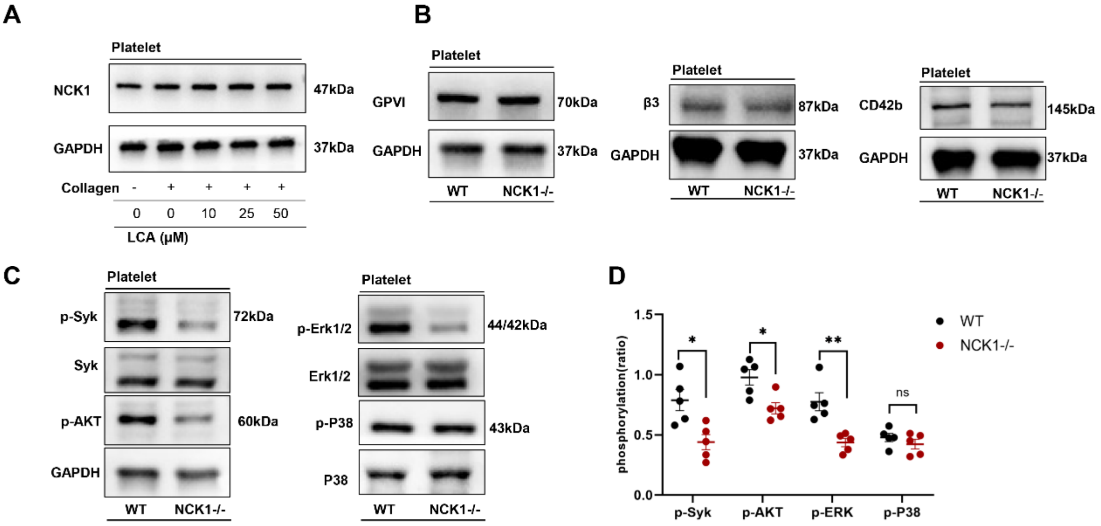

Supplemental Figure 5. The mechanism of NCK1 participating in platelet activation.
(A) Human platelets were pretreated with LCA (0 μM, 10 μM, 25 μM, or 50 μM) stimulated with collagen, and lysed with lysis buffer for immunoblotting. The expression of NCK1 was detected by western blot. (B) The platelets of WT and NCK1−/− mice were extracted to determine the expression level of GPVI, Integrin β3, CD42b/GPIb. (C, D) The platelets of WT and NCK1−/− mice were stimulated with collagen, and lysed with lysis buffer for immunoblotting. We used the primary antibodies, including p-SYK, p-AKT, p-ERK1/2, and p-P38; N = 5. Data are presented as the mean ± standard error of mean; one-way ANOVA; *P < 0.05, **P < 0.01, ***P < 0.001, ****P < 0.0001, NS indicates no significance.

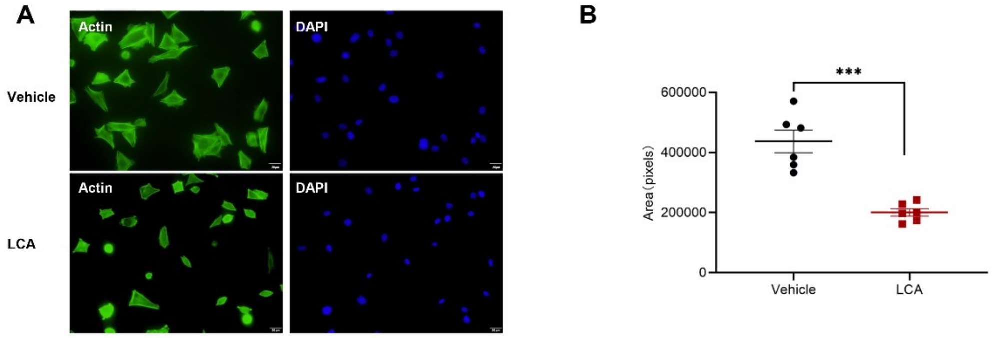

Supplemental Figure 6. LCA inhibited αIIbβ3-CHO cells spreading.

(A) Representative immunofluorescence images of actin in αIIbβ3-CHO cells spreading on immobilized fibrinogen in the absence or presence of LCA. (B) The summary of spreading area in two groups; N = 6. Data are presented as the mean ± standard error of mean; Unpaired t-test, ***P < 0.001. αIIbβ3, glycoprotein IIb/IIIa; LCA, lithocholic acid.

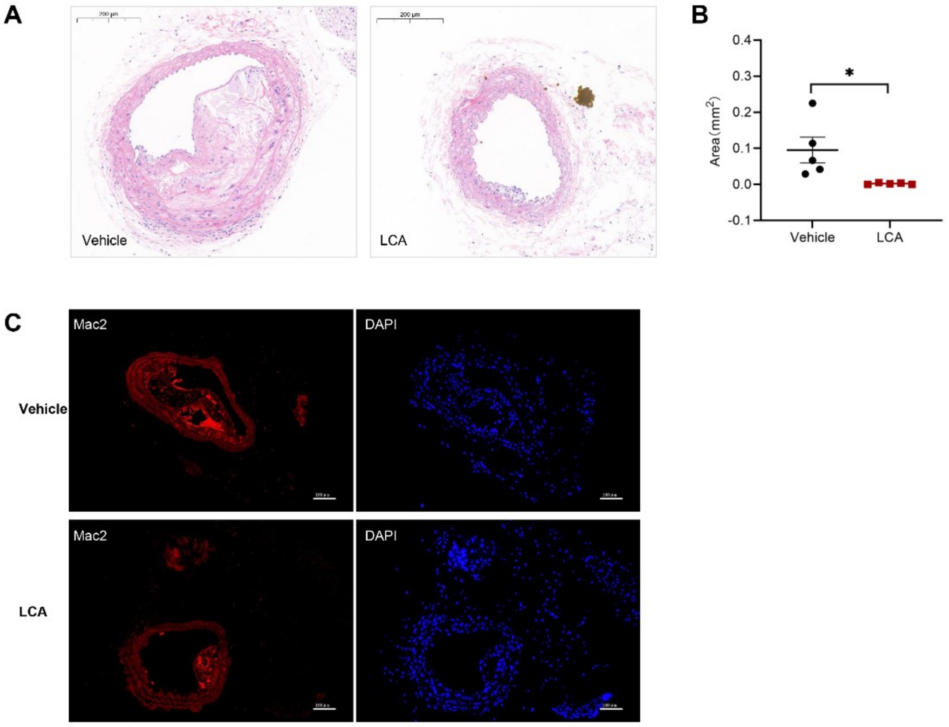

Supplemental Figure 7. LCA reduced atherosclerotic plaque formation.

(A) Representative HE staining images of carotid atherosclerotic plaques and (B) quantification of plaque area between experimental groups. (C) Representative immunofluorescence images of carotid artery plaque stained for macrophages (Mac-2+, red) among LCA-treated mice and vehicle-treated mice. Data are presented as the mean ± standard error of mean; one-way ANOVA; *P < 0.05, **P < 0.01, ***P < 0.001, ****P < 0.0001, NS indicates no significance.
